# 3D Structure and Processing Methods Direct the Biological Attributes of ECM-Based Cardiac Scaffolds

**DOI:** 10.1038/s41598-019-41831-9

**Published:** 2019-04-03

**Authors:** Yael Efraim, Beth Schoen, Sharbel Zahran, Tzila Davidov, Gleb Vasilyev, Limor Baruch, Eyal Zussman, Marcelle Machluf

**Affiliations:** 1 Faculty of Biotechnology & Food Engineering, Technion – Israel Institute of Technology, Haifa, 3200003 Israel; 2 Faculty of Mechanical Engineering, Technion – Israel Institute of Technology, Haifa, 3200003 Israel

## Abstract

High hopes are held for cardiac regenerative therapy, driving a vast research effort towards the development of various cardiac scaffolds using diverse technologies and materials. Nevertheless, the role of factors such as fabrication process and structure in determining scaffold’s characteristics is yet to be discovered. In the present study, the effects of 3D structure and processing method on cardiac scaffolds are addressed using three distinct scaffolds made through different production technologies from the same biomaterial: decellularized porcine cardiac extracellular matrix (pcECM). pcECM patch, injectable pcECM hydrogel, and electrospun pcECM scaffolds were all proven as viable prospective therapies for MI, thus generally preserving pcECM beneficial properties. Yet, as we demonstrate, minor differences in scaffolds composition and micro-morphology as well as substantial differences in their mechanical properties, which arise from their production process, highly affect the interactions of the scaffold with both proliferating cells and functional cells. Hence, the rates of cell attachment, survival, and proliferation significantly vary between the different scaffolds. Moreover, major differences in cell morphology and alignment as well as in matrix remodeling are obtained. Overall, the effects revealed herein can guide a more rational scaffold design for the improved cellular or acellular treatment of different cardiac disease scenarios.

## Introduction

The field of cardiac tissue engineering has evolved tremendously in the last decade^1^, with the development of different approaches to fabricate 3D cardiac tissue constructs, including injectable hydrogels^2^, cell sheets^3^, and cardiac patches^4,5^. The developed scaffolds are generally designed to support cell recruitment, adherence and growth, and possibly to provide the needed mechanical support to the cardiac tissue^6^. Nevertheless, the broad and diverse outcomes of different studies, which were obtained for specific scaffolds manufactured using different technologies, raise the crucial need to gain a profound understanding of the contribution of other important factors such as fabrication process, modality, and structure to scaffold characteristics and performance. Fabrication as well as isolation processes, which aim at granting the scaffold with different advantages (specifically when biological scaffolds are considered), can change it in terms of preservation of the original material or of micro- and macro-structural properties, and consequently, in terms of mechanical characteristics. These changes in composition, stiffness, micro, and macrostructure could be crucial to scaffold success, as they can highly affect cell behavior, cell-scaffold interactions, and signal transduction as well as the scaffold integration with the damaged tissue^7–10^. Pore size, for example, affects infiltration and proliferation of host cells^11^. In addition, scaffold stiffness was shown to affect scaffold remodeling^12^, as well as cell migration and differentiation^13^. Therefore, to address these effects, different 3D scaffolds, made of the same material, using different technologies, should be investigated thus pinpointing the effect of each parameter on the therapeutic outcome.

Our group and others have exploited the decellularized porcine cardiac ECM (pcECM) for cardiac regenerative therapy^14–17^, as it is considered a promising biomaterial that best mimics the natural cell microenvironment^18–20^. With its unique structure and composition (collagens, glycosaminoglycans, etc.) and its biodegradability and bioactivity, decellularized ECM scaffolds can play an active complex role in regulating cell behavior, and influence cell survival, proliferation, migration, shape, and function^21^. Using our patented decellularization protocol (WO 2006095342A2), we produced pcECM which served as the raw material for the development of three distinct scaffolds: (1) decellularized pcECM patch (D-patch)^14,22^, (2) novel electrospun pcECM patch (ES-patch)^23^, and (3) pcECM-based hydrogel (hydrogel)^24^. Each one of these scaffolds was demonstrated to be a viable platform for prospective cardiac regenerative therapy, thus significantly preserving the composition, micro-morphology, and bioactivity of the native cardiac ECM. Nevertheless, these constructs were produced using distinct technologies; they differ in their 3D end product, and thus may differ in their mode of application and regenerative effects. While the soft hydrogel can be non-invasively injected into the damaged tissue, and combined with either cells or factors, the cardiac patches application requires invasive surgical procedures^2^. Furthermore, while the electrospun pcECM scaffold enables the controllable and reproducible design of scaffold characteristic, the D-patch relay on the preservation of the inherent characteristics of the original ECM material^14,25^.

In the current work we, therefore, aim to reveal the effect of the 3D structure and processing method on scaffolds derived from the same original material: decellularized pcECM. We have addressed the scaffolds composition, micro-morphology and mechanical properties, and evaluated the effect of these factors on cell-scaffold interactions of both proliferating cells (stem cells) and functional cells (cardiomyocytes) in terms of scaffold remodeling, cell viability, alignment, and functionality. Understanding the effects of the different fabrication methods and structures on scaffold functionality could not only provide fundamental insights on cell-scaffold interactions but also lead the field of tissue engineering to a more rational material and scaffold design, which could, in turn, facilitate their prospective clinical application.

## Results

### pcECM-based scaffolds production

**Figure 1:**
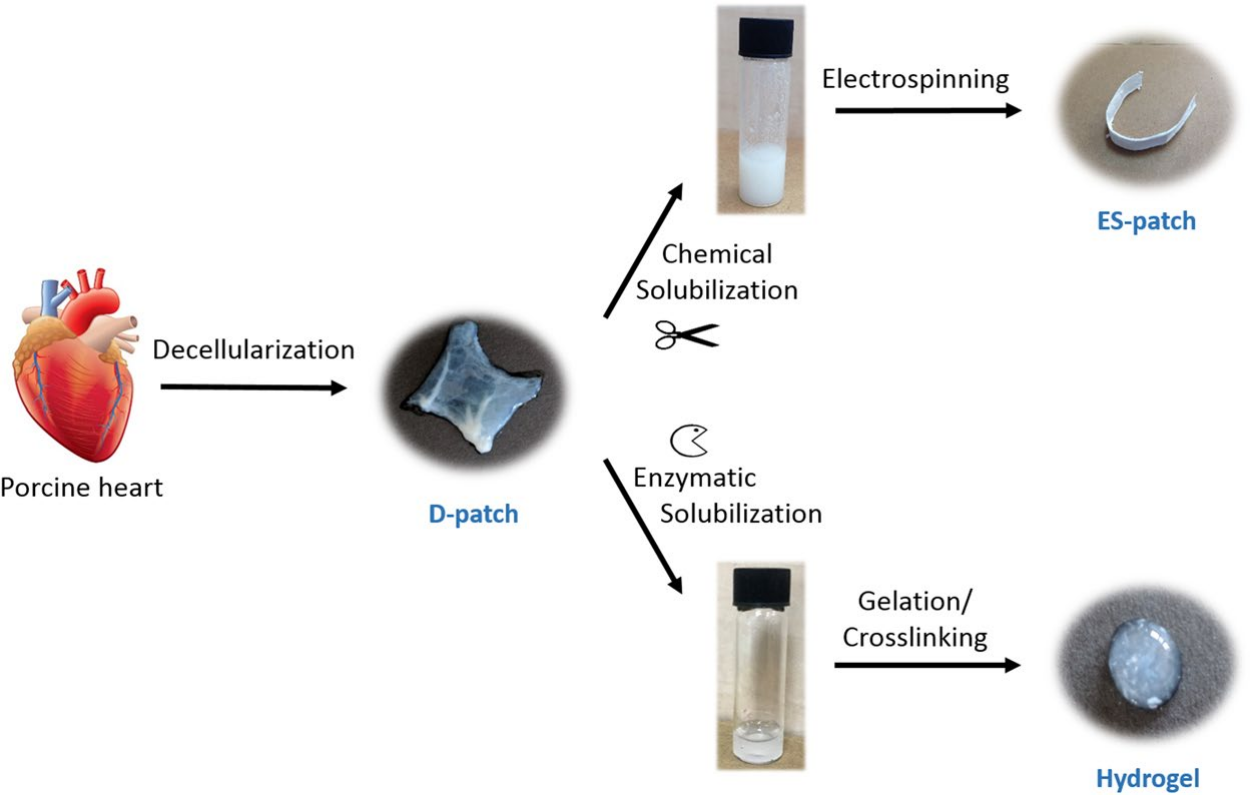
Scaffolds preparation. Illustration of the three fabrication methodologies used to create the pcECM-based scaffolds, including decellularization, solubilization, gelation, and electrospinning. (Heart drawing: dreamstime.com, image 49749529 by Andegraund548).

Thin slices of pcECM were produced using our unique decellularization procedure, which avoids the use of the strong detergent SDS and thus highly preserves ECM components^14^. These ECM slices served as the basis for our three distinct pcECM-based scaffolds: D-patches were cut to the desired size directly from the pcECM using steel punch. ES-patches were produced through the electrospinning of pcECM solubilized in HFIP, and the hydrogel was produced using pepsin digestion of pcECM in aqueous solution and gelation at 37 °C (Fig. 1).

### pcECM-based scaffolds composition

**Figure 2:**
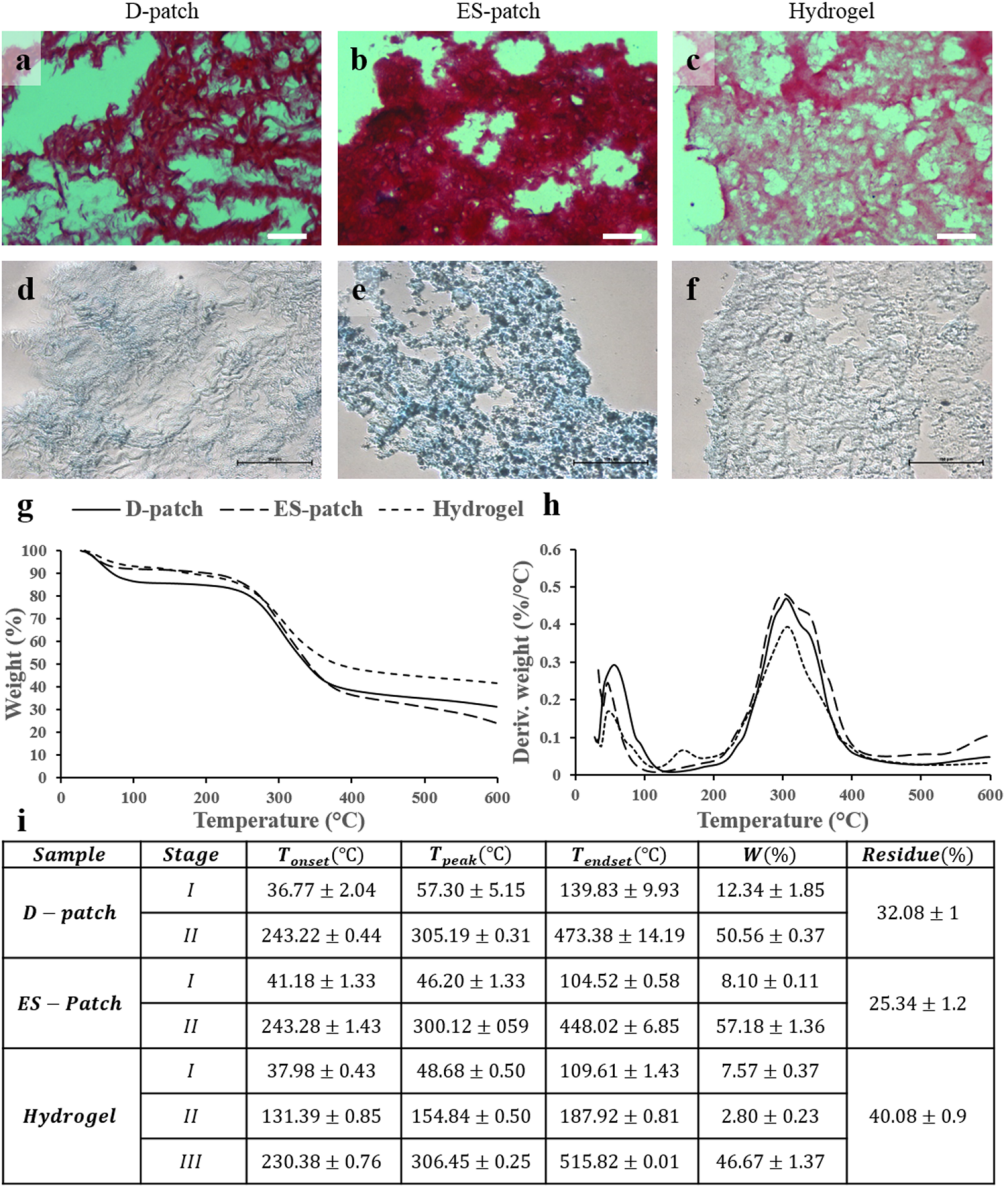
Scaffolds composition. Visualization of collagen and GAG content of each pcECM-based scaffold by PicroSirius red (**a**–**c**, scale bar 40 µm) and alcian blue (**d**–**f**, scale bar 100 µm), respectively. D-patch (**a**,**d**), ES-patch (**b**,**e**) and hydrogel (**c**,**f**). Thermal gravimetric analysis (TGA, n = 3) curves, presented as % weight (**g**), Derivative of weight (**h**), and a summarizing table (i). *p < 0.05 compared to D-patch.

Staining the three scaffolds; D-patch, ES-patch, and Hydrogel with PicroSirius red, which stains collagen red, and Alcian Blue, which stains glycosaminoglycans blue, demonstrates that the scaffolds are mostly comprised of collagen with the presence of GAGs. The highest concentration of collagen and GAGs are visible in the ES-patch (Fig. 2a–f). To understand the thermal behavior, as an indication of the collagen degradation in the different scaffolds^26,27^, we have performed thermogravimetric analysis (TGA). The thermal degradation characteristics are presented in Fig. 2g–i where T_onset_ is the temperature at which the thermal degradation begins, T_peak_ is the temperature at which the thermal degradation is maximal, T_endset_ is the temperature at which the process is complete, and W (%) is the weight percentage loss during each stage. As shown in Fig. 2i, for all scaffolds, the first degradation process that starts below 100 °C is attributed to the loss of bound water, when the D-patch presents higher T_peak_ and T_endset_ for this stage. The second degradation process (third in the hydrogel) is attributed to the collagenous components and is quite similar in all the scaffolds with an earlier onset and latest endset for the hydrogel. Also, only the hydrogel exhibited a third degradation process which can be seen in the additional peak at ~150 °C (Stage II for hydrogel in Fig. 2i). Moreover, the hydrogel presents the highest residual material with nearly 40% left, and the ES-patch the lowest with about 25% residues.

**Figure 3:**
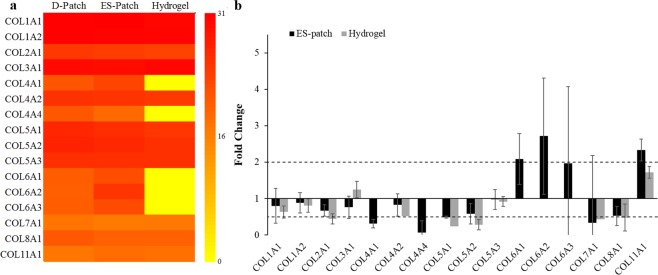
Scaffolds protein composition (n = 4). Protein abundance presented as heatmap for each scaffold. Scale represents log2 of protein abundance and ranges from low (yellow) to high (red) protein abundance (**a**). Fold change of a specific protein content in the hydrogel and ES-patch compared to the D-patch (**b**). Significant change (>2 or <0.5)^42^ is indicated by the dashed line.

To further support these results, and to assess their collagenous composition, the three scaffolds were subjected to proteomic analysis. Primarily, all three scaffolds comprised the α1 and α2 chains of collagen type 1 in similar quantities; with the total amount of collagen type 1 being the largest single collagen type followed by collagen type 3, and all other types of collagen being significantly less abundant (Fig. 3a). Evaluating the fold-change in both the ES-patch and hydrogel vs. the D-patch (Fig. 3b) revealed that the relative quantities of the major collagen types (I and III) were highly preserved, and there was no significant fold-change between their percentages in the D-patch, the ES-patch or the hydrogel. Other collagen types exhibited different degrees of preservation, demonstrated through significantly lower percentages of collagen 4 α-1 and α-4, and a significantly higher percentage of collagen 6 α-2 in the ES-patch. Collagen 6, however, was not identified in the hydrogel. Less significant changes were also exhibited in the lower percentages of collagen types 2, 5 and 8 in the hydrogel, and in the higher percentage of collagen 11 α-1 obtained in the ES-patch.

### Molecular structure and microstructure of the different pcECM-based scaffolds

**Figure 4:**
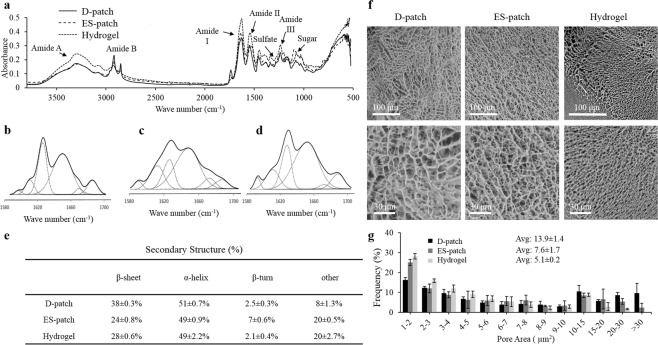
Scaffolds structure. FTIR spectra of the three scaffolds (**a**, n = 3). The secondary structure of scaffolds proteins determined by Fourier deconvolution of each scaffold’s amid I band, D-patch (**b**), ES-patch (**c**), and hydrogel (**d**). Summary of the secondary structures of the scaffolds’ proteins is presented in table e. Cryo-SEM images of D-patch, ES-patch, and hydrogel (**f**, n = 3). Scale bar 100 µm upper panel, and 30 µm lower panel. Pore size analysis presented as the frequency (%) of pore area for each scaffold (**g**). *p < 0.05 compared to D-patch.

FTIR analysis was used to confirm the presence of different bonds such as; amide bonds, sulfates, and sugars (GAGs, Fig. 4a). Bands corresponded to amide A (3274 cm^−1^), amid B (2915 cm-1) and the three main bands of the collagen fingerprint: amide I (1613 cm^−1^) due to carbonyl stretching, amide II (1539 cm^−1^) due to vibration on the plane of N-H bond, and C-N stretching, and amide III (1231 cm^−1^) due to C-N stretching and N-H deformation^28–30^. The individual secondary structures of the proteins were evaluated through deconvolution and curve fitting of the amide I band^28,31–33^. It can be seen that the deconvolution methods used provided a good fit to the data (Fig. 4b–d). The table in Fig. 4e summarizes the extent of each secondary structure in the three scaffolds. While all scaffold exhibited similar percentages of α-helix structure (~50%), the processed scaffolds—hydrogel and ES-patch—contained less β-sheet and more unidentified structures (others). In addition, we see an increase in β-turn structure in the ES-patch compared to the others.

Scaffolds microstructure and morphology were characterized using scanning electron microscopy (SEM) and image analyses, which revealed that all scaffolds had a fiber mesh morphology, typical for pcECM (Fig. 4f). However, while thicker fibers and larger pores (13.9 + 1.4 μm^2^) characterized the D-patch, the ES-patch was characterized by thinner fibers and significantly smaller pores (7.6 + 1.7 μm^2^), and the hydrogel was characterized by the thinnest fibers and smallest pores (5.1 + 0.2 μm^2^, p < 0.001, Fig. 4f,g).

### Mechanical properties

**Figure 5:**
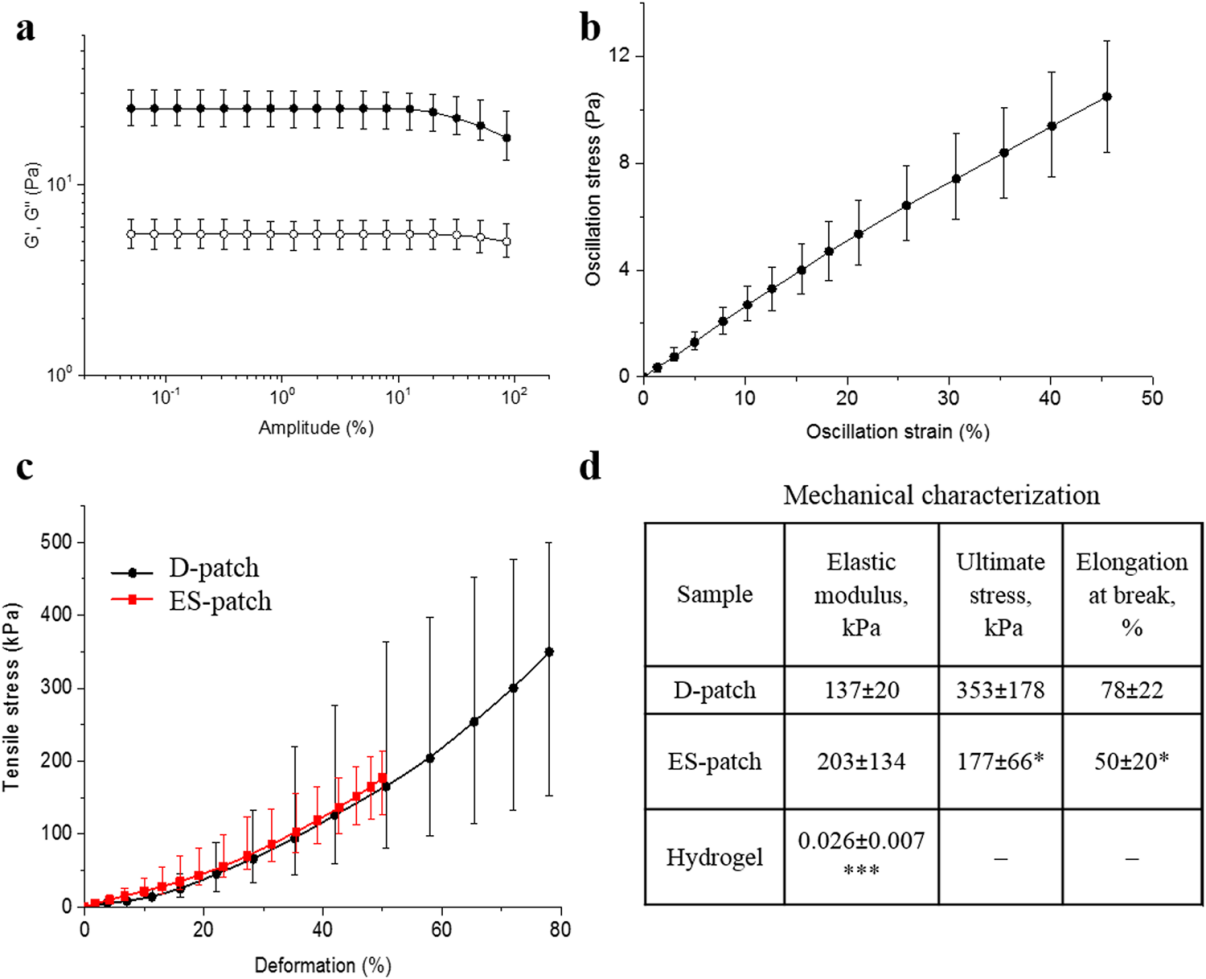
Scaffolds mechanical properties (n = 4). Storage (filled symbols) and loss (open symbols) moduli versus amplitude of oscillatory deformation of the hydrogel at 1 Hz and 37 °C (**a**). Stress-strain curve recalculated from the data of oscillatory shear deformation tests at 37 °C (**b**). Stress-Strain curves for the D-patch and ES-patch at 37 °C (**c**). Summarizing table of all scaffolds mechanical characteristics (**d**). *p < 0.1, ***p < 0.0001 compared to D-patch.

To compare the mechanical properties of the distinct pcECM-based scaffolds, Young’s modulus of the hydrogel was calculated from its oscillatory shear deformation measurements (Fig. 5a,b), and Young’s modulus of the D-patch and the ES-patch were calculated from tensile tests (Fig. 5c). Comparison of Young’s modulus of the scaffolds showed that the modulus of the hydrogel is lower by almost 4 orders of magnitude than the one of the D-patch (26 Pa and 137 kPa, respectively). ES-patch, on the other hand, was characterized in a non-significant, slightly higher modulus than the D-patch (203 kPa, Fig. 5d).

### Cell-scaffold interactions - proliferating cells

**Figure 6:**
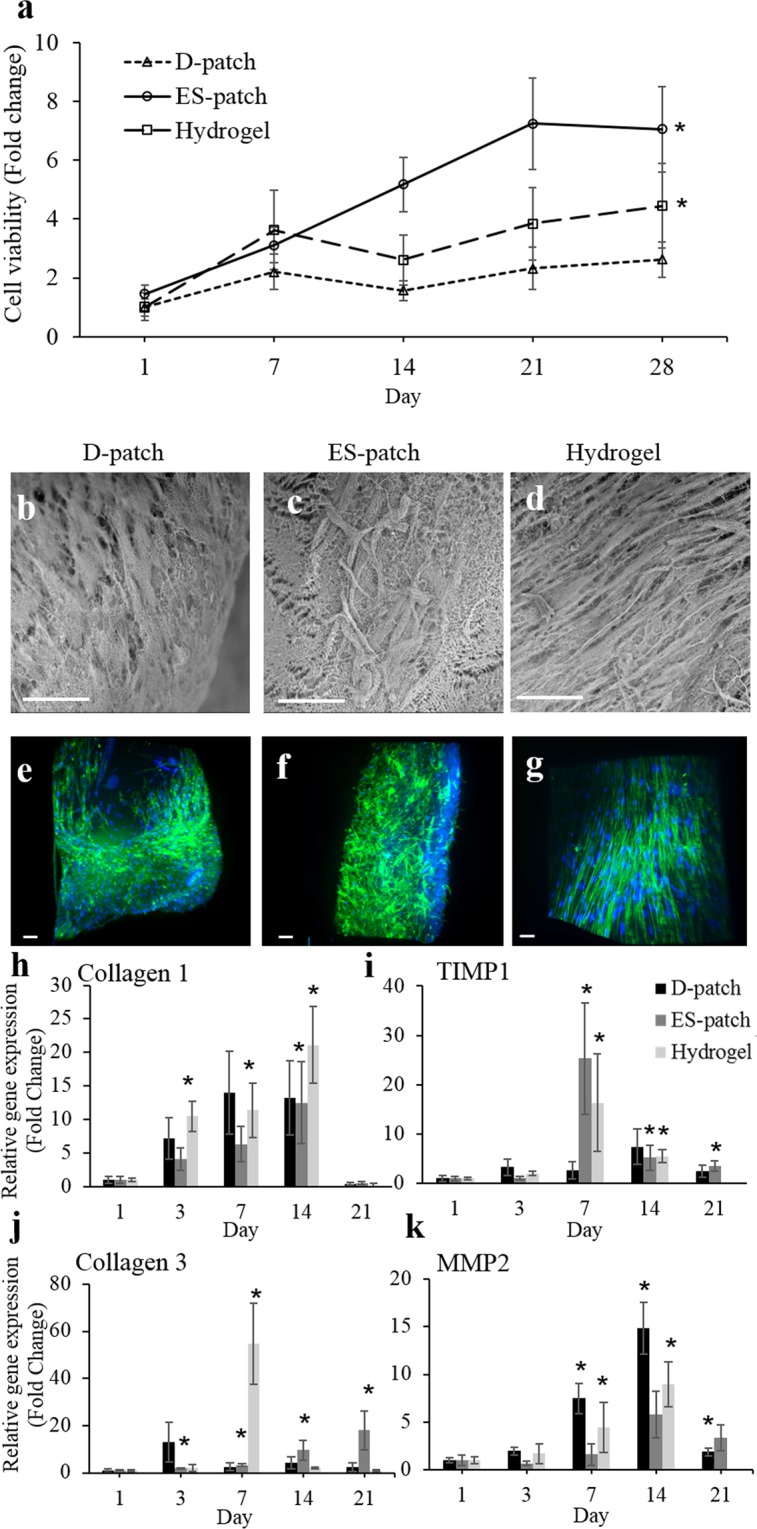
MSCs cultivation on pcECM-based scaffolds (n = 5). Cell viability on pcECM-based scaffolds, over four weeks, presented as fold change from D-patch at day 1 (**a**). *p < 0.05 compared to D-patch. Cryo-SEM (**b**–**d**) and LSFM (**e**–**g**) imaging of hMSCs seeded D-patch (**b**,**e**), ES-patch (**c**,**f**), and hydrogel (**d**,**g**). Scale bar 80 µm (**b**–**d**) and 100 µm (**e**–**g**). Green: phalloidin (Actin), blue: Hoechst (DNA). Expression of ECM remodeling genes by hMSCs cultured on the three scaffolds, relative to day 1 (*p < 0.05). Collagen I (**h**) Tissue inhibitor of metalloproteinases type 1 (TIMP1) (**i**) Collagen III (**j**) Matrix metalloproteinase-2 (MMP2) (**k**).

The ability of our pcECM-based scaffolds to support proliferating cells’ culture and growth was addressed using human bone marrow mesenchymal stem cells (hMSCs). Cells were seeded and cultured on the D-patch, ES-patch, and hydrogel for four weeks and their viability was compared. As shown in Fig. 6a, all three scaffolds supported the adherence of hMSCs, which continued to proliferate throughout the experiment reaching a 2.6-fold increase in cell number on the D-patch, a 4.4-fold increase on the hydrogel, and a 7-fold increase on the ES-patch (two-way ANOVA, p < 0.05). The morphology of the cultured hMSCs was imaged by SEM (Fig. 6b–d), and light-sheet fluorescent microscopy (Fig. 6e–g) 28 days post-seeding, demonstrating that hMSCs had populated all the pcECM-based scaffolds. Interestingly, hMSCs seeded on the hydrogel had aligned to a single direction, forming a tissue-like structure. This alignment was also seen in the D-patch, to some extent, however not on the ES-patch.

Analysis of ECM remodeling related gene expression was used to compare the cells ability to remodel the different pcECM-based scaffolds. In all seeded scaffolds, collagen I expression increased by up to 10–20 folds 3, 7, and 14 days post seeding when compared to the basal levels. This elevated expression decreased by day 21 (Fig. 6h). The maximal increase in collagen III expressions was observed in day 3 for the D-patch (13 fold), day 7 for the hydrogel (55 fold) and day 21 for the ES-patch (18 fold, Fig. 6i). TIMP1 (collagenase inhibitor) maximal expression was observed at day 7 for the hydrogel and ES-patch (16 and 25 fold respectively) and at day 14 for the D-patch (7 fold, Fig. 6j). The expression of MMP2, an important matrix metalloproteinase, had increased at day 7 in all three scaffolds (1.5–7.5 fold) and reached maximal expression at day 14 (5–15 fold, Fig. 6k).

### Cell-scaffold interactions - cardiac cells

**Figure 7:**
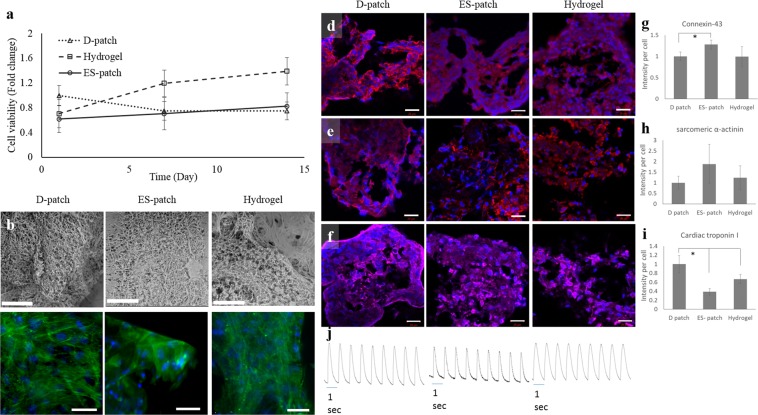
hiPS-CM cultivation on pcECM-based scaffolds. Cell viability on pcECM-based scaffolds: D-patch, ES-patch, and hydrogel, over 14 days (n = 5) (**a**). Cryo-SEM imaging (**b**, n = 3) of hiPS-CM seeded D-patch, ES-patch, and hydrogel. Scale bar 100 µm. LSFM imaging (**c**, n = 3) of hiPS-CM seeded D-patch, ES-patch, and hydrogel; green: phalloidin (Actin), blue: Hoechst (DNA). Scale bar 50 µm. Cardiac markers expressed by hiPS-CM cultured on D-patch, ES-patch, and hydrogel for 14 days (**d**–**f**). Blue: Hoechst (DNA), red: Connexin-43 (**d**) or sarcomeric α-actinin (SAA, **e**), pink: cardiac troponin I (cTn1, **f**). Scale bar 100 µm. The intensity of cardiac markers expression was quantified, normalized to the number of cells and expressed relative to the D-patch (**g**–**I**, *p < 0.05). Confocal line scan images showing changes in intracellular Ca^2+^ in a Fluo-4 loaded of hiPS-CM seeded D-patch, ES-patch, and hydrogel 14 days post seeding (**j**). The whole cell Ca^2+^ transient in 1 Hz induced pacing frequency is shown.

To assess the pcECM-based scaffolds ability to support the cultivation of a “beating” cardiac cell as well as its contraction and electrical coupling, human induced pluripotent stem cell-derived cardiomyocytes (hiPS-CM) were seeded on the three pcECM-based scaffolds and followed for 14 days. Viability measurements revealed adherence and survival of the cells on all three scaffolds. However, D-patch seeded cells presented higher adherence (p < 0.05), and in terms of proliferation, the growth remained static on the ES-patch, decreased on the D-patch (p < 0.01) and increased on the hydrogel (Fig. 7a, p < 0.01). Cryo-SEM and LSFM imaging of the hydrogel-seeded hiPS-CM revealed a more organized morphology with visible orientation compared to the cells seeded on the D-patch and ES-patch (Fig. 7b,c). The hiPS-CM on all the scaffolds were positively stained for the cardiac markers; cardiac troponin I, sarcomeric alpha-actinin and connexin-43 (Fig. 7d–i), with different expression profiles on each scaffold. Moreover, they started beating spontaneously within the first 24 hr of culture (Supplementary Videos 1–3). This contractile functioning and cell-cell electrical coupling were also demonstrated using [Ca^2+^] imaging at day 14. As seen, whole-cell [Ca^2+^] transients during pacing (using field stimuli) at 1 Hz rates were achieved, displayed as a line-scan tracing (Fig. 7j).

## Discussion

Among the biomaterials suggested for cardiac tissue engineering, ECM—and pcECM in particular—stands out as the promising choice due to its resemblances to the native tissue and the presence of bioactive molecules that drive tissue homeostasis and regeneration^15,18,34,35^. Different studies, including ours^14,16,17,22,24^, have reported the use of pcECM as a natural patch or as an injectable scaffold, alone or combined with different polymers and moieties^14–16,24,36–40^. Recently, we have also developed a technology for the electrospinning of pcECM, which allowed the production of a novel electrospun whole pcECM scaffold^23^. While all these pcECM-based scaffolds were proven as viable prospective therapies for myocardial infarction (MI), the questions regarding the contribution of important factors such as fabrication process, modality, and structure to the 3D scaffold’s characteristics and performance remain to be answered. Although using the same material, the production of different pcECM-based platforms may result in different properties, such as composition, structure, physical and mechanical characteristics, thus leading to different biological properties, different interactions with residing cells, and different therapeutic efficacy. Therefore, in the current study, we have explored three scaffolds, which are based on pcECM, and fabricated using different technologies. Our aim was to evaluate these scaffolds in terms of composition, micro-morphology and mechanical properties, and assess the effect of these factors on cell-scaffold interactions.

In terms of scaffolds composition, we could confirm that both collagen and glycosaminoglycans are still major constituents of all three scaffolds. Moreover, we demonstrated that the different production processes used to create each scaffold did not affect the relative quantities of the most abundant components of the cardiac ECM: collagens type I and III. These components generally provide the architectural support for muscle cells and myocardial function^41^, and their ratio is significant for achieving the necessary mechanical properties within the heart. The remaining collagen types, which are found in the ECM in lower quantities, were differently preserved in each scaffold production methodology. In relevance to the fold-change, it is generally accepted that larger than a 2-fold increase or 0.5-fold decrease in protein abundance is significant^42^. The most obvious changes in abundance were for collagen type VI, which was not identified at the hydrogel. However, studies by Luther *et al*. have demonstrated that the knockout of collagen type VI actually improves cardiac function and remodeling following myocardial infarction^43,44^. Thus, the missing collagen VI in our engineered scaffold has the potential to benefit its function. Collagen molecules degradation through the production process—assessed through thermogravimetric analysis—was shown to differ between the different pcECM-based scaffolds. While all scaffolds presented similar T_peak_ of collagen degradation, suggesting similar composition of the collagenous component, the ES-patch presented lower T_endset_ and residues that can be attributed to some extent to the chemical degradation throughout its solubilization in an organic solvent^45^. The hydrogel, on the other hand, presented lower T_onset_ and higher T_endset_ with ~40% residues. The earlier onset can be attributed to the enzymatic degradation needed for the pcECM solubilization, and the higher T_endset_ and higher residue can suggest improved stability due to the crosslinking with genipin^46,47^. In addition, we witnessed higher T_peak_ of stage I in the D-patch, indicating a stronger connection to bound water molecule in the original non-soluble material. The additional stage of degradation seen in the Hydrogel can be attributed to the pepsin enzyme, which is added to the solubilization process^27^. This non-active enzyme is present in the hydrogel scaffold but is not covalently bonded to the collagenous main structure.

Following these studies, we addressed, using protein conformational analysis, the structural components of the proteins. Our results revealed that the main α-helix structure was preserved among the three scaffolds (~50%), however, other secondary structures were altered in the processed scaffolds, hydrogel and ES-patch. While in the hydrogel, the decreased β-sheet structure can be attributed to the effect of pepsin digestion on proteins as previously demonstrated by Yang *et al*.^48^, in the ES-patch, changes can be attributed to the electrospinning process, which was previously shown to cause major conformational changes in the secondary structure of different proteins^31,49,50^.

Addressing the scaffolds’ microstructure, our results demonstrated that while the fibrous structure that characterizes natural cardiac ECM is preserved in all scaffolds, matrix density and porosity varied between the different scaffolds. The reduction in pore size from the D-patch to the ES-patch can be explained through the slightly thinner and denser original electrospun fibers, which upon wetting self-assembled into a denser matrix of thinner fibers and smaller pores, respectively^23^. The large decrease in the hydrogel pore size is, however, a direct result of the enhanced crosslinking due to the genipin crosslinker and the available hydrogen bonding sites^51^. Since the hydrogel is primarily water, the hydrogen bonding sites are more readily accessible than in the patch scaffolds. These production parameters can be, naturally, controlled and scaffolds of larger pore size can be tailored upon need. An additional contribution to the reduced pore size of the processed scaffolds may be attributed to the altered secondary structures that are known to affect the collagen fibrillar organization^52^.

Scaffold cytocompatibility is crucial for its function and integration with the cardiac tissue when implanted with cells or acellular. To assess the cell-supporting capabilities of our pcECM-based scaffolds, we have used hMSCs and human induced pluripotent stem cells derived cardiomyocytes (hiPS-CMs), two popular candidates for cardiac therapy. MSCs can potentially differentiate into the major cell types composing the myocardium: cardiac endothelial cells, cardiomyocytes, and myofibroblasts^53^. Moreover, owing to their safeness and immunomodulatory properties, MSCs are used in many biomedical applications and clinical studies^53^. To assess the scaffold’s ability to support cardiac cellular functionality, contractility, and electrical coupling hiPS-CMs were utilized. Their advantage lies in their high availability, and the potential use of human autologous cells with no ethical concerns. From our studies, it is evident that both hMSCs and hiPS-CMs can adhere and survive on all pcECM-based scaffolds. Nevertheless, the number of adhered cells, maximal cell density, cell morphology and their crosstalk with the scaffold differed between the chosen cells and between the scaffolds. While the hMSC similarly adhered to all the scaffolds, their highest proliferation was obtained when seeded on the ES-patch and the lowest proliferation was obtained when seeded on the D-patch. The high cell proliferation on the ES-patch was not accompanied, though, with the elongated cell morphology and aligned tissue-like structure that characterized their culture on the hydrogel and, to some extent, on the D-patch. These different cell behaviors on the different pcECM scaffolds result from the scaffolds’ microstructure and mechanical properties, as the ECM stiffness, compliance, and microstructure are known to exert mechanical cues that—through transcription regulation—affect cell shape as well as growth, survival, and differentiation^54–56^. Hence, stiffer scaffolds were shown to promote MSCs proliferation^13,57,58^, which is in accordance with the highest proliferation that was obtained on the ES-patch. Nevertheless, it contradicts the lower hMSCs numbers obtained on the D-patch compared to the softer hydrogel (Fig. 6). These lower numbers can be attributed, however, to the larger pores characterizing this scaffold, which can lead to lower proliferation rates^59^. Moreover, the softer, more compliant, hydrogel might have altered the cytoskeletal tension and actin structure, leading to aligned, narrower cell shape.

Cell-scaffold interactions and scaffold bioactivity can also be characterized by the way the cells remodel their surrounding matrix. When examining the expression profiles of ECM-remodeling genes^14,24^, it can be concluded that the three pcECM-based scaffolds—like natural pcECM^60,61^—are bioactive, thus dynamic crosstalk is held between the hMSC and each of the scaffolds. However, while all the scaffolds promoted high expression levels of collagen I, collagen III expression is clearly higher on the hydrogel. Collagen III contributes to scaffold compliance while collagen I contributes to scaffold stiffness and rigidity^62^. Considering the extremely lower Young’s modulus of the hydrogel, this more elastic scaffold might be driving the cells to further increase its compliance and allow tissue-like alignment. The more catabolic activity of the cells on the D-patch, compared to the ES-patch scaffold and the hydrogel could be due to the D-patch thickness, which promotes ECM digestion to allow cell penetration into its deeper layers. These *in vitro* changes in gene expression are, however, temporary, as without additional physiological signals the cells eventually reach a steady state.

When culturing hiPS-CMs on the three pcECM-based scaffolds, significantly higher adherence was obtained on the D-patch. Within seven days, though, the cell number had decreased, reaching the levels of the other scaffolds initial adherence. Fully differentiated hiPS-CMs hold limited ability to proliferate. Nevertheless, the number of cells cultured on the hydrogel increased along the 14 days of the study. Our differentiation procedure, modified from Burridge *et al*.^63^, is known to yield 80–95% beating cardiomyocytes, with a small portion of proliferating cells (Ki67+ cells). It is, therefore, concluded that these proliferating cells are accountable for the increased cell numbers in the hydrogel, which is considerably softer than the ES-patch and D-patch. Studies have shown that softer platforms promote stemness retention^64,65^. Moreover, the organ-specific differentiation of stem cells was demonstrated to benefit a mechanical stiffness that mimics the one of the respective tissue^54,55^. Hence, the hydrogel—which exhibited lower Young’s modulus than the heart tissue—led to reduced cardiac differentiation rates. Notably, all scaffolds enabled synchronized beating when seeded with hiPS-CMs and were stained positive for the cardiac markers, thus confirming that pcECM-based scaffolds, regardless of their processing technology, can support the intact synchronized electrical activity of the heart.

Altogether, for the first time, we have presented a comprehensive “same material-different technology” study, revealing the effect of the fabrication process and modality on scaffold characteristics and function *in vitro*. While the soft pcECM hydrogel—the scaffold with the minimally invasive delivery potential—preserved the major components of the ECM and their ratio, minor types of collagen were not preserved during the enzymatic processing, and the reconstruction of a part of the β-sheet secondary structures was altered. The more compliant 3D ECM structure was denser and promoted substantial remodeling by the seeded cells, thus supporting cell alignment in a tissue-like structure. Notably, this ECM modality has also promoted the proliferation of cells that were not differentiated into cardiomyocytes, which can lead to the creation of successful composite tissue on the one hand but increase the risk for teratoma formation using hiPSC-derived cells on the other. The D-patch, a thicker, porosive and most natural construct, supported cell adherence and viability, however not proliferation in these culture conditions. Moreover, it mainly promoted a catabolic remodeling activity. The ES-patch—the most technologically controllable scaffold—presented similar stiffness to native ECM and did not support cell alignment. However, it promoted the highest cell proliferation, which might suggest its advantage in cell recruitment and the production of a highly-populated graft.

## Conclusion

Holding a tremendous potential for cardiac tissue engineering, pcECM-based scaffolds generally preserve pcECM beneficial properties, such as collagenous composition, fibrous isotropic structure, bioactivity, biocompatibility and the ability to support cellular culture and function. However, when considering the treatment of different cardiac disease scenarios, with or without cells of different types, one should bear in mind the unique properties of each scaffold—as revealed herein.

## Methods

### Scaffolds production

All three scaffolds, the D-patch, the ES-patch scaffold, and hydrogel, originated from the same material—decellularized pcECM—which was produced through the decellularization procedure we have previously published^14^. In brief, left ventricular tissues, isolated from healthy commercial slaughter-weight pigs and cut into 3 mm-thick slices, were decellularized using two cycles of alternating hyper/hypo-tonic NaCl solutions, enzymatic treatment using trypsin, and washes with Triton-X-100. Tissues from at least 3 animals were processed at each batch. The decellularized pcECM was then processed as follows to produce the different pcECM scaffolds.

D-patch preparation: Decellularized slices of pcECM were cut to the desired shape using a steel punch, sterilized and preserved in PBS solution supplemented with Pen-Strep (1%) and Fungizone (0.4%) at 4 °C^14^.

### ES-patch preparation

Decellularized, sterilized pcECM was frozen in liquid nitrogen, crushed in a cryogenic tissue grinder (BioSpec, Bartlesville, OK, USA), and subsequently lyophilized. Dry pcECM was dissolved in hexafluoroisopropanol (HFIP) to a concentration of 0.05 g mL^−1^ and homogenized using ZrO beads in a Precellys® 24-bead homogenizer (Bertin Technologies, Rockville, MD, USA) at 6000 rpm until the solution appeared homogeneous. The solution was filtered and polyethylene oxide (PEO, 600 kg mol^−1^) was added to obtain a final solution of 0.1 mass %. The pcECM/PEO solution was electrospun using a custom-built electrospinning device as previously reported^23^. PEO was removed from the matrix by washing in an aqueous solution.

### Hydrogel preparation

The decellularized pcECM was frozen in liquid nitrogen, crushed and lyophilized to a dry fine powder. The powder was solubilized (10 mg ml^−1^) in HCl (0.01 M) using sonication (1 min), followed by enzymatic digestion using pepsin (1 mg ml^−1^). The solution was then adjusted to pH = 5 with NaOH and kept cold (4 °C). To crosslink the solubilized pcECM, genipin (Sigma-Aldrich, 0.01 gr genipin/gr ECM) was added, and the samples were plated at 37 °C for 3 hrs. Following, PBS was added to prevent dehydration^24^.

### Scaffolds composition analyses

Proteomic analysis: Protein composition analysis was performed at The Smoler Protein Research Center, Technion – Israel Institute of Technology. In brief, samples (n = 4 scaffolds, for each scaffold) were digested by trypsin, and the resulting peptides were analyzed by LC-MS/MS. After which, the peptide mix was fractionated by HPLC and electro-sprayed onto an ion-trap mass spectrometer, in order to determine the proteins’ mass. The peptides were further fragmented by collision-induced dissociation and analyzed again. Peptides were analyzed and identified using Proteome Discoverer^TM^ software (Thermo-Scientific) against the porcine part of the UniProt database. If a protein was uncharacterized in the database, a basic alignment search tool (BLAST) was performed on the UniProt database that is used to find regions of local similarity between sequences to identify members of the same gene family.

Histology: Scaffolds (n = 3) were fixed in PFA (4%) for 20 minutes, washed in PBS, frozen in Tissue-Tek® OCT compound (Sakura, Netherlands), and cross-sectioned into slices (10 μm) on glass slides for staining. Slides were fixed in cold MeOH (4 °C) for 20 min prior to staining. After fixation, slides were washed in DDW 3–5 times to remove all the OCT compound and stained with PicroSirius Red Stain Kit (Abcam) for collagen and Alcian Blue (Merck Millipore) for GAGs according to the manufacturer instructions. Images were taken using Light microscopy (Nikon TE-2000).

### Structure and properties

Protein secondary structure: All scaffolds (n = 3) were lyophilized prior to FTIR analysis. FTIR spectra were then recorded using a Thermo 6700 FTIR instrument, equipped with a Smart iTR Attenuated Total Reflectance (ATR) diamond plate, in the wave-number range 500–3500 cm^−1^ (64 scans at a resolution of 4 cm^−1^). Data were evaluated using OMNIC^TM^ series software (version 8, Thermo Scientific). The secondary structures of collagen proteins were determined using the spectral curve fitting of amide I region (1,700–1,600 cm^−1^) and band narrowing of the spectrum by Fourier deconvolution followed by peak choosing and fitting. Six peaks were chosen and assigned to a specific secondary structure according to the literature for the original D-patch followed by peak fitting for the other two scaffolds.

### Thermal degradation

Thermal gravimetric analysis (TGA) data were obtained using a TGA-Q5000 system (TA Instruments, USA). Lyophilized samples (n = 3) were heated from room temperature at a rate of 20 °C min^−1^ under a nitrogen atmosphere to a final temperature of 600 °C. Data were analyzed using TA Universal Analysis Software (TA Instruments, DE, USA).

### Scanning Electron Microscopy (SEM)

Wet scaffolds (n = 3) were visualized using a Phenom ProX desktop SEM (PhenomWorld, Eindhoven, Netherlands), equipped with a temperature controlled sample holder (Deben, UK Ltd., Suffolk, UK). Samples were mounted on aluminum stubs using tissue freezing medium (Ted Pella, Inc., Ca, USA), and cooled to −24 °C. Images were captured at 15 kV accelerating voltage. Pore size analyses were conducted using the Porometric software (PhenomWorld).

### Mechanical characterization

DHR-2 rheometer was used to apply oscillatory shear deformation under torsion mode at the strain control mode. Parallel plate geometry (PP) with a diameter of 40 mm was used when testing the hydrogel (n = 4 biological replicates) at the frequency of 1 Hz at 37 °C. Young’s modulus was calculated from the oscillatory shear deformation^66^. A custom-made horizontal tensile machine equipped with a controlled-temperature bath and a Sensotec® 2 N load cell (model 31/1435-03) was used to carry out tensile tests for the wet D-patch and ES-patch (n = 4 biological replicates for each scaffold). The stretching rate was 3.0 mm min^−1^. The force generated during the stretching process was recorded. ES-patches were cut into strips with the size of 20 × 4 mm; the distance between the clamps was set to 12.3 mm. The thickness was measured for each specimen individually at 3 different points, and the averaged value was calculated. D-patches were cut into samples of 4 mm in width, and the distance between the clamps was set to 10.0 mm. The thickness was measured in the middle of the sample, and the width was measured again after the sample was clamped. All samples were soaked for 1 minute in DW at 37 °C before testing.

### Cell studies

Scaffold preparation: D-patches were cut into circles (D = 0.18 cm) and placed into 96 well plates. Fresh media was added and plates were placed in a humidified incubator (37 °C, 5% CO_2_) for one day, then the media was replaced and cells were seeded. ES-patch were cut into circles (D = 0.18 cm) and placed into 96 well plates. Plates were placed in a humidified incubator (37 °C, 5% CO_2_). After an hour the media was replaced and returned to the incubator for at least 12 hours before the cells were seeded. 96 well plates were coated with 30 μl Hydrogel, allowed to gel in 37° for 3 hr. Following, fresh media was added, and the plate was kept in a humidified incubator (37 °C, 5% CO_2_) for at least 12 hours before cells were seeded.

### Cell culture

hMSCs (Lonza, Basel, Switzerland) were seeded on the different scaffolds (10,000 per scaffold) and cultured for 4 weeks. hMSCs were cultured in αMEM, supplemented with FCS (10%), pen-strep (1%), fungizone (0.4%), and basic fibroblast growth factor (5 ng mL^−1^). The medium was replaced every second day. hiPSC-CM were generated according to a published protocol^63^, from an individual hiPSC batch. hiPSC-CM were seeded on day 17–21 of differentiation on the different scaffolds (30,000 per well) and cultured for 14 days. The hiPSC-CM were cultured in RPMI Medium and supplemented with 2% B-27 minus insulin (Gibco). Media was replaced every second day. Cell viability was evaluated using the AlamarBlue™ reagent (AbD Serotec, Kidlington, UK), according to the manufacturer protocol. These studies included 5 biological replicates (5 scaffolds of each type) of which 2 technical replicates were sampled.

### Lightsheet Fluorescent Microscopy (LSFM)

All cell-seeded scaffolds were stained with Hoechst 33258 (for DNA), and FITC-phalloidin. Scaffolds were then embedded within low-melting agarose gel and inserted into a 1 mL tube. The sample was analyzed by LSFM (Lightsheet Z.1, Zeiss). Multichannel Z-stack images were obtained to visualize the morphology of the stained cells.

### MSCs remodeling of pcECM-based scaffolds

The expression of ECM remodeling-related genes by hMSCs seeded on the pcECM-based scaffolds was quantitatively studied for 21 days, by real-time RT-PCR. The hMSCs were seeded on the scaffolds as described above. Total RNA was isolated using Tri-reagent (Sigma-Aldrich) according to the manufacturer instructions, and reverse-transcribed in a PTC-200 PCR cycler using a Verso^TM^ cDNA kit (Thermo-Scientific). These studies included 5 biological replicates (5 scaffolds of each type) of which 3 technical replicates were taken.

The following primers were used:

5′-TACAGCGTCACTGTCGATGGC-3′ and 5′-TCAATCACTGTCTTGCCCCAG-3′ for collagen Iα1; 5′-AATTTGGTGTGGACGTTGGC-3′ and 5′-TTGTCGGTCACTTGCACTGG-3′ for collagen III α1^67^; 5′-TTGACGGTAAGGACGGACTC-3′ and 5′-ACTTGCAGTACTCCCCATCG-3′ for MMP2; 5′-TACTTCCACAGGTCCCACAA-3′ and 5′-ATTCCTCACAGCCAACAGTG-3′ for TIMP1^68^; and 5′-CAACAGCGACACCCACTCCT-3′ and 5′-CACCCTGTTGCTGTAGCCAAA-3′ for glyceraldehyde 3-phosphate dehydrogenase (GAPDH) as an intrinsic housekeeping gene control. Reactions were run on the StepOnePlus system and analyzed using StepOne software v. 2.2.2 (Applied Biosystems).

### Histology

Cell-seeded scaffolds were processed on paraffin blocks, sectioned (5 µm, Shandon Finesse 325 Microtome) and stained. Cardiac troponin I, sarcomeric alpha-actinin, and connexin-43 cardiac markers were used for immunostaining hiPSC-CM seeded scaffolds according to the manufacturer’s protocol. Slides were visualized using LSM-700 confocal microscope, and the expression of cardiac markers was quantified using Imaris software and at least 5 images of each type of scaffold.

### Ca^2+^ imaging (n = 3)

Ca^2+^ imaging was performed according to a previously published protocol^69^. Briefly, cells were loaded with 5 mM of fluo-4 fluorescent Ca^2+^ indicator (Molecular Probes) in the presence of Pluronic F-127 (Molecular Probes) to allow the recording of intracellular Ca^2+^-transients. For pacing, scaffolds were plated on a 35-mm optical plate (Matek) with field stimulation electrodes (RC-37FS; Warner Instruments) and paced using a stimulus isolation unit (SIU-102; Warner Instruments), by applying 5 ms-suprathreshold bipolar stimulation pulses up to 50 mA. Intracellular Ca^2+^-transients were recorded using a Zeiss laser-scanning confocal imaging system (Fluo-view; Olympus) mounted on an upright BX51WI Olympus microscope equipped with a X60 water objective. Data were analyzed utilizing a MatLab-based custom-written software.

### Statistical analysis

The data are expressed as mean ± standard deviation. Analysis of variance (ANOVA) was used for comparison using JMP 14 from SAS software. p < 0.05 was considered significant unless otherwise mentioned.

## Supplementary information

Supplementary Video 1

Supplementary Video 2

Supplementary Video 3

## References

[1] Hirt MN, Hansen A, Eschenhagen T (2014). Cardiac Tissue Engineering. Circ. Res..

[2] Hasan A (2015). Injectable Hydrogels for Cardiac Tissue Repair after Myocardial Infarction. Adv. Sci..

[3] Haraguchi, Y. *et al*. Cell Sheet Technology for Cardiac Tissue Engineering. in *Cardiac Tissue Engineering: Methods and Protocols* (eds Radisic, M. & Black, L. D. III) 139–155 10.1007/978-1-4939-1047-2_13 (Springer New York, 2014). 10.1007/978-1-4939-1047-2_13 25070334

[4] Prabhakaran MP, Venugopal J, Kai D, Ramakrishna S (2011). Biomimetic material strategies for cardiac tissue engineering. Materials Science and Engineering C.

[5] Zhao G, Zhang X, Lu TJ, Xu F (2015). Recent advances in electrospun nanofibrous scaffolds for cardiac tissue engineering. Adv. Funct. Mater..

[6] Camci-Unal G, Annabi N, Dokmeci MR, Liao R, Khademhosseini A (2014). Hydrogels for cardiac tissue engineering. NPG Asia Mater.

[7] Chang, H.-I. & Wang, Y. Cell Responses to Surface and Architecture of Tissue Engineering Scaffolds. *Regen*. *Med*. *Tissue Eng*. *- Cells Biomater*. 569–588, 10.5772/21983 (2011).

[8] Sun Y, Chen CS, Fu J (2012). Forcing Stem Cells to Behave: A Biophysical Perspective of the Cellular Microenvironment. Annu. Rev. Biophys..

[9] Nguyen AT, Sathe SR, Yim EKF (2016). From nano to micro: topographical scale and its impact on cell adhesion, morphology and contact guidance. J. Phys. Condens. Matter.

[10] Harley BAC (2008). Microarchitecture of three-dimensional scaffolds influences cell migration behavior via junction interactions. Biophys. J..

[11] Rnjak-Kovacina J (2011). Tailoring the porosity and pore size of electrospun synthetic human elastin scaffolds for dermal tissue engineering. Biomaterials.

[12] Petersen A, Joly P, Bergmann C, Korus G, Duda GN (2012). The Impact of Substrate Stiffness and Mechanical Loading on Fibroblast-Induced Scaffold Remodeling. Tissue Eng. Part A.

[13] Breuls RGM, Jiya TU, Smit TH (2008). Scaffold Stiffness Influences Cell Behavior: Opportunities for Skeletal. Tissue Engineering. Open Orthop. J..

[14] Eitan Y, Sarig U, Dahan N, Machluf M (2010). Acellular cardiac extracellular matrix as a scaffold for tissue engineering: *in vitro* cell support, remodeling, and biocompatibility. Tissue Eng Part C Methods.

[15] Robinson KA (2005). Extracellular matrix scaffold for cardiac repair. Circulation.

[16] Sarig U (2012). Thick acellular heart extracellular matrix with inherent vasculature: Potential platform for myocardial tissue regeneration. Tissue Eng. Part A.

[17] Sarig U (2015). Pushing the Envelope in Tissue Engineering: *Ex Vivo* Production of Thick Vascularized Cardiac Extracellular Matrix Constructs. Tissue Eng. Part A.

[18] Elçin MP (2016). and A. D. and S. O. and A. E. E. and Y. M. Clinical applications of decellularized extracellular matrices for tissue engineering and regenerative medicine. Biomed. Mater..

[19] Fitzpatrick LE, McDevitt TC (2015). Cell-derived matrices for tissue engineering and regenerative medicine applications(1). Biomater. Sci..

[20] Moroni F, Mirabella T (2014). Decellularized matrices for cardiovascular tissue engineering. Am J Stem Cells.

[21] Frantz C, Stewart KM, Weaver VM (2010). The extracellular matrix at a glance. J. Cell Sci..

[22] Sarig U (2016). Natural myocardial ECM patch drives cardiac progenitor based restoration even after scarring. Acta Biomater..

[23] Schoen B (2017). Electrospun Extracellular Matrix: Paving the Way to Tailor-Made Natural Scaffolds for Cardiac Tissue Regeneration. Adv. Funct. Mater..

[24] Efraim Y (2017). Biohybrid cardiac ECM-based hydrogels improve long term cardiac function post myocardial infarction. Acta Biomater..

[25] Wainwright JM (2010). Preparation of cardiac extracellular matrix from an intact porcine heart. Tissue Eng. Part C. Methods.

[26] León-Mancilla BH, Araiza-Téllez MA, Flores-Flores JO, Piña-Barba MC (2016). Physico-chemical characterization of collagen scaffolds for tissue engineering. J. Appl. Res. Technol..

[27] Rusu AG, Popa MI, Lisa G, Vereştiuc L (2015). Thermal behavior of hydrophobically modified hydrogels using TGA/FTIR/MS analysis technique. Thermochim. Acta.

[28] Yang H, Yang S, Kong J, Dong A, Yu S (2015). Obtaining information about protein secondary structures in aqueous solution using Fourier transform IR spectroscopy. Nat. Protoc..

[29] Nagai T, Suzuki N, Tanoue Y, Kai N (2012). Collagen from Tendon of Yezo Sika Deer (&lt;i&gt;Cervus nippon yesoensis&lt;/i&gt;) as By-Product. Food Nutr. Sci..

[30] Silva Júnior ZS (2015). Effect of papain-based gel on type I collagen–spectroscopy applied for microstructural analysis. Sci. Rep..

[31] Stephens JS, Chase DB, Rabolt JF (2004). Effect of the Electrospinning Process on Polymer Crystallization Chain Conformation in Nylon-6 and Nylon-12. Macromolecules.

[32] Chadefaux C, Le Hô A-S, Bellot-Gurlet L, Reiche I (2009). Curve-fitting Micro-ATR-FTIR studies of the Amide I and II bands of Type I collagen in archaeological bone materials. e-PRESERVATION Sci..

[33] Barth A (2007). Infrared spectroscopy of proteins. Biochimica et Biophysica Acta - Bioenergetics.

[34] Chen WCW (2016). Decellularized zebrafish cardiac extracellular matrix induces mammalian heart regeneration. Sci. Adv..

[35] Benders KEM (2013). Extracellular matrix scaffolds for cartilage and bone regeneration. Trends in Biotechnology.

[36] Grover GN, Rao N, Christman KL (2014). Myocardial matrix-polyethylene glycol hybrid hydrogels for tissue engineering. Nanotechnology.

[37] Singelyn JM (2009). Naturally derived myocardial matrix as an injectable scaffold for cardiac tissue engineering. Biomaterials.

[38] Marsano A (2013). The effect of controlled expression of VEGF by transduced myoblasts in a cardiac patch on vascularization in a mouse model of myocardial infarction. Biomaterials.

[39] Miyagi Y (2011). Biodegradable collagen patch with covalently immobilized VEGF for myocardial repair. Biomaterials.

[40] Zammaretti P, Jaconi M (2004). Cardiac tissue engineering: Regeneration of the wounded heart. Current Opinion in Biotechnology.

[41] Pauschinger M (1999). Dilated Cardiomyopathy Is Associated With Significant Changes in Collagen Type I/III ratio. Circulation.

[42] Mann M, Kelleher NL (2008). Precision proteomics: the case for high resolution and high mass accuracy. Proc. Natl. Acad. Sci. USA.

[43] Luther DJ (2013). Knockout of type VI collagen preserves mitochondrial structure and function following myocardial infarction. FASEB J..

[44] Luther DJ (2012). Absence of Type VI Collagen Paradoxically Improves Cardiac Function, Structure and Remodeling Following Myocardial Infarction. Circ. Res..

[45] Iafisco M, Foltran I, Sabbatini S, Tosi G, Roveri N (2012). Electrospun Nanostructured Fibers of Collagen-Biomimetic Apatite on Titanium Alloy. Bioinorg. Chem. Appl..

[46] Duan S, Kai T, Saito T, Yamazaki K, Ikeda K (2014). Effect of Cross-Linking on the Mechanical and Thermal Properties of Poly(amidoamine) Dendrimer/Poly(vinyl alcohol) Hybrid Membranes for CO(2) Separation. Membranes (Basel)..

[47] Pati F, Adhikari B, Dhara S (2010). Isolation and characterization of fish scale collagen of higher thermal stability. Bioresour. Technol..

[48] Yang, Y. *et al*. Secondary Structure and Subunit Composition of Soy Protein *In Vitro* Digested by Pepsin and Its Relation with Digestibility. *Biomed Res*. *Int*. **2016** (2016). 10.1155/2016/5498639 PMC4889807 27298825

[49] Stephens JS (2005). Effects of Electrospinning and Solution Casting Protocols on the Secondary Structure of a Genetically Engineered Dragline Spider Silk Analogue Investigated via Fourier Transform Raman Spectroscopy. Biomacromolecules.

[50] Huang X (2014). Tunable Structures and Properties of Electrospun Regenerated Silk Fibroin Mats Annealed in Water Vapor at Different Times and Temperatures. J. Nanomater..

[51] McKegney M, Taggart I, Grant MH (2001). The influence of crosslinking agents and diamines on the pore size, morphology and the biological stability of collagen sponges and their effect on cell penetration through the sponge matrix. J. Mater. Sci. Mater. Med..

[52] Shoulders MD, Raines RT (2009). Collagen structure and stability. Annu. Rev. Biochem..

[53] Appasani, K. & Appasani, R. K. *Stem Cells &amp; Regenerative Medicine*, 10.1007/978-1-60761-860-7 (Springer, 2011).

[54] Gattazzo F, Urciuolo A, Bonaldo P (2014). Extracellular matrix: A dynamic microenvironment for stem cell niche. Biochimica et Biophysica Acta - General Subjects.

[55] Mammoto A, Mammoto T, Ingber DE (2012). Mechanosensitive mechanisms in transcriptional regulation. J. Cell Sci..

[56] Dufort CC, Paszek MJ, Weaver VM (2011). Balancing forces: Architectural control of mechanotransduction. Nature Reviews Molecular Cell Biology.

[57] Park JS (2011). The Effect of Matrix Stiffness on the Differentiation of Mesenchymal Stem Cells in Response to TGF-β. Biomaterials.

[58] Wang L-S, Boulaire J, Chan PPY, Chung JE, Kurisawa M (2010). The role of stiffness of gelatin–hydroxyphenylpropionic acid hydrogels formed by enzyme-mediated crosslinking on the differentiation of human mesenchymal stem cell. Biomaterials.

[59] Bružauskaitė I, Bironaitė D, Bagdonas E, Bernotienė E (2016). Scaffolds and cells for tissue regeneration: different scaffold pore sizes—different cell effects. Cytotechnology.

[60] Badylak SF, Freytes DO, Gilbert TW (2009). Extracellular matrix as a biological scaffold material: Structure and function. Acta Biomater..

[61] Zhang J (2016). Perfusion-decellularized skeletal muscle as a three-dimensional scaffold with a vascular network template. Biomaterials.

[62] Kwak H-B (2013). Aging, exercise, and extracellular matrix in the heart. J. Exerc. Rehabil..

[63] Burridge PW (2014). Chemically defined generation of human cardiomyocytes. Nat Meth.

[64] Ghasemi-Mobarakeh L (2015). Structural properties of scaffolds: Crucial parameters towards stem cells differentiation. World J. Stem Cells.

[65] Gilbert PM (2010). Substrate elasticity regulates skeletal muscle stem cell self-renewal in culture. Science.

[66] Malkin, A. Y. & Isayev, A. I. *Rheology Concepts*, *Methods*, *and Applications*. *Rheology Concepts*, *Methods*, *and Applications*, 10.1016/B978-1-895198-49-2.50014-1 (2012).

[67] Schmidt A, Lorkowski S, Seidler D, Breithardt G, Buddecke E (2006). TGF-β1 generates a specific multicomponent extracellular matrix in human coronary SMC. Eur. J. Clin. Invest..

[68] Mu X, Urso ML, Murray K, Fu F, Li Y (2010). Relaxin regulates MMP expression and promotes satellite cell mobilization during muscle healing in both young and aged mice. Am. J. Pathol..

[69] Itzhaki I (2011). Calcium handling in human induced pluripotent stem cell derived cardiomyocytes. PLoS One.

